# Role of information in preparing men for transrectal ultrasound guided prostate biopsy: a qualitative study embedded in the ProtecT trial

**DOI:** 10.1186/s12913-015-0729-z

**Published:** 2015-02-28

**Authors:** Julia Wade, Derek J Rosario, Joanne Howson, Kerry N L Avery, C Elizabeth Salter, M Louise Goodwin, Jane M Blazeby, J Athene Lane, Chris Metcalfe, David E Neal, Freddie C Hamdy, Jenny L Donovan

**Affiliations:** School of Social and Community Medicine, University of Bristol, 39 Whatley Road, Clifton, Bristol, BS8 2PS UK; Academic Urology Unit, Royal Hallamshire Hospital, University of Sheffield, Sheffield, S10 2JF UK; Protect study Urology Research, Royal Hallamshire Hospital, Sheffield, S10 2JF UK; Oncology Centre, Addenbrooke’s Hospital, Hills Road, Cambridge, CB2 0QQ UK; Nuffield Department of Surgical Sciences, University of Oxford, John Radcliffe Hospital, Headley Way, Oxford, OX3 9DU UK

**Keywords:** Biopsy, Cancer, Patient information, Prostate, Qualitative methods

## Abstract

**Background:**

The histological diagnosis of prostate cancer requires a prostate needle biopsy. Little is known about the relationship between information provided to prepare men for transrectal ultrasound guided biopsy (TRUS-Bx) and how men experience biopsy. The objectives were a) to understand men’s experiences of biopsy as compared to their expectations; and b) to propose current evidence-based information for men undergoing TRUS-Bx.

**Methods:**

Between February 2006 and May 2008, 1,147 men undergoing a standardised 10-core transrectal ultrasound guided biopsy protocol under antibiotic cover following a PSA 3.0-19.9 ng/ml in the Prostate Testing for Cancer and Treatment (ProtecT) trial, completed questionnaires about biopsy symptoms. In this embedded qualitative study, in-depth interviews were undertaken with 85 men (mean age 63.6 yrs, mean PSA 4.5 ng/ml) to explore men’s experiences of prostate biopsy and how the experience might be improved. Interview data were analysed thematically using qualitative research methods. Findings from the qualitative study were used to guide selection of key findings from the questionnaire study in developing a patient information leaflet preparing men for biopsy.

**Results:**

Although most men tolerated TRUS-Bx, a quarter reported problematic side-effects and anxiety. Side effects were perceived as problematic and anxiety arose most commonly when experiences deviated from information provided. Men who were unprepared for elements of TRUS-Bx procedure or its sequelae responded by contacting health professionals for reassurance and voiced frustration that pre-biopsy information had understated the possible severity or duration of pain/discomfort and bleeding. Findings from questionnaire and interview data were combined to propose a comprehensive, evidence-based patient information leaflet for TRUS-Bx.

**Conclusions:**

Men reported anxiety associated with TRUS-Bx or its side-effects most commonly if they felt inadequately prepared for the procedure. Data from this qualitative study and the previous questionnaire study have been used to propose an updated, comprehensive evidence-based set of information for men undergoing TRUS-Bx.

**Electronic supplementary material:**

The online version of this article (doi:10.1186/s12913-015-0729-z) contains supplementary material, which is available to authorized users.

## Background

Prostate cancer (PCa) is the second most common cause of cancer death in European men [1] with an incidence rate of 214 cases per 1000 [2]. Biopsy is required for histological diagnosis of PCa. Moreover, as active surveillance (AS) becomes increasingly accepted as an alternative to radical treatment for localised PCa [3], scheduled re-biopsy is also used as a monitoring tool. Transrectal ultrasound guided biopsy (TRUS-Bx) is therefore one of the most commonly performed urological procedures with over one million procedures performed each year in Europe and the United States (US) [4]. A recent systematic review of complications arising from TRUS-Bx found that, although symptoms of minor bleeding or urinary complications are common, they do not usually require intervention; infective complications, though uncommon, can be serious and require rapid intervention [4].

Population screening for PCa using prostate specific antigen (PSA) testing is currently not recommended, due to ongoing controversies regarding the net balance of benefits against harms, including potential harms of prostate biopsy [5-7]. Instead, European Association of Urology (EAU) guidelines on PCa recommend that opportunistic PSA screening should be offered to the well-informed man [8]. Men in the United Kingdom (UK) are provided with information about the advantages and disadvantages of PSA testing, prostate biopsy and prostate cancer treatments to facilitate shared or informed decision-making [9]. This process of shared and informed decision-making requires that patients be given accurate and comprehensive information about prostate biopsy and its sequelae [10].

The *P*rostate *B*iopsy *E*ffects (ProBE) study [11], is the largest prospective cohort study to date of morbidity arising from TRUS-Bx, based on men receiving a standardized biopsy protocol as part of the ProtecT (*Pro*state *te*sting for *c*ancer and *T*reatment) randomised controlled trial (RCT). The ProBE study investigated men’s experiences of physical sequelae and impact on health-related quality of life (HRQL), including healthcare use [11] and anxiety [12], using data from self-report questionnaires. The ProBE study [11] found that the prevalence of post-biopsy symptoms was higher as compared to previous reports [13,14]. In addition, although symptoms were rated as a ‘major’ or ‘moderate’ problem by a minority only (pain = 7.3%, fever = 5.5%, haematuria = 6.2%, heamatochezia = 2.5%, heamoejaculate = 26.6%), more than one quarter (27.1%) reported one or more symptoms as problematic (i.e. a ‘moderate’ or ‘major’ problem) during the 35 days post-biopsy [11]. Further analysis found that men experiencing symptoms as a ‘major’ or ‘moderate’ problem were more likely to report increased anxiety at seven days post biopsy prior to the biopsy outcome being known and regardless of the ultimate biopsy result [12]. What remained unclear was why around one quarter of men experienced symptoms as problematic and how associated anxiety arose.

The present study addresses these issues. It reports a qualitative in-depth interview study embedded within the ProBE/ProtecT studies (a) to understand men’s experiences of biopsy as compared to expectations before biopsy; and (b) to propose current evidence-based information for men undergoing TRUS-Bx with a view to minimising anxiety associated with problematic symptoms [12].

## Methods

### ProBE/ProtecT study designs

The ProBE study investigated impacts of TRUS-Bx in a population invited for PSA testing (for details see Rosario et al. [11]). Briefly, from February 2006 to May 2008, 1,147 (65%) of 1,753 ProtecT study participants aged 50–69 years, with a raised PSA result (3.0 -19.9 ng/ml) were invited for digital rectal examination (DRE), repeat PSA test and standardized 10-core TRUS-Bx under antibiotic cover [11]. Men returned purpose designed questionnaires assessing the physical harms of biopsy at seven and 35 days post biopsy and the Hospital Anxiety and Depression Scale [15] assessing the psychological status at the time of initial PSA test, at time of biopsy and at seven days (before biopsy result was known) and 35 days post biopsy (after biopsy result was known). Further details of data collection and analysis in the questionnaire study are reported elsewhere [11,12]. A pre-study questionnaire completed by each ProBE study centre showed that seven out of eight study centres routinely administered local anaesthetic prior to biopsy. All men invited to join the ProBE study received patient information leaflets (PILs) on the ProBE and ProtecT studies, as well as the relevant local hospital TRUS-Bx PILs and explanations from staff carrying out the procedure (see Additional file 1 for a sample PIL used by one study centre). Ethical approval was obtained from Trent Multicentre Research Ethics Committee, UK. All participants gave informed consent.

### Participants in the qualitative study

Three groups of participants were recruited to the qualitative investigation of the TRUS-Bx experience. Using maximum variation sampling to include men with a wide range of characteristics and biopsy experiences, 45 ProBE study participants with a range of ages, socio-economic backgrounds and various biopsy outcomes were invited for interview. Experience of post-biopsy infection was not captured in this sample; therefore five additional men with confirmed infection were sampled from ProBE study participants. Within the ProtecT study, a further 53 men purposively sampled to achieve maximum variation sampling were invited for interviews investigating their experiences of participating in the study [16] and including questions about their experience of biopsy.

### Interviews

In-depth qualitative interviews were conducted after biopsy result was known in the ProBE study by KNLA (Table 1, A1-A33) and JW (Table 1, A34i-A38i) a median of 10 and 18 weeks following biopsy, and within the ProtecT study, by JW, CES and JLD (Table 1, B39-B85) a median of 41 weeks after biopsy. Interviews were by telephone or face-to-face in each man’s preferred location. Interviews were semi-structured using a topic guide (see Issues covered by Topic Guide) to elicit expectations and actual experiences of TRUS-Bx and its sequelae and reflect on how negative impacts might be mitigated, whilst simultaneously allowing men to raise individual issues.

**Table 1:** **Characteristics of in-depth interview study participants, N = 85**

	**ProBE/ProtecT participants N = 38**		**ProtecT participants N = 47**	**All participants**
	**No infection (N = 33, A1-A33)**	**Infection (i) (N = 5, A34i-A38i)**	**(N = 47, B39—B85)**	**(N = 85)**
**Age at time of first biopsy: mean (SD)**	64.3 (4.9)	60.8 (4.9)	63.5 (4.5)	63.6 (4.7)
**Employment status N (%)**				
Full time	14	5	24	43
Not working	18	0	20	38
Part time	0	0	0	0
Not specified/missing	1	0	3	4
**Ethnicity, N (%)**				
White	33	5	46	84
Other	0	0	1	1
**Centre, N (%)**				
1	3	0	0	3
2	1	0	9	10
3	16	3	30	49
4	2	0	8	10
5	4	0	0	4
6	3	1	0	4
7	2	1	0	3
8	2	0	0	2
**Initial PSA result ng/ml, median (Interquartile range)**	6.0 (3.7 to 13.0)	4 (3.4 to 4.7)	4.3 (3.5 to 6.7)	4.5 (3.5 to 7.2)
**Biopsy result**			(Final biopsy)	
Benign	12	1	0	13
Localized cancer	12	4	47	63
Advanced cancer	9	0	0	9
**Number of biopsies at time of interview**				
1	33	5	35	73
2	0	0	10	10
3	0	0	2	2
**Interview type**				
Telephone	18	5	0	23
Face to face	15	0	47	62
**Timing of interview N weeks post-biopsy** Median (range) mean	10 (3–138) 33	18 (10–72) 35	41 (9–75)* 41	40 (3–138) 39
**Treatment of infection**				
Hospital admission	n/a	3	n/a	3
Family physician	n/a	2	1**	3
**Cancer treatment**				
Radical prostatectomy	^−^	-	17	17
Radical radiotherapy	-	-	15	15
Active monitoring	-	-	15	15
Other	-	-	0	0

*calculated from date of most recent biopsy if more than one biopsy took place**1 man was prescribed antibiotics having consulted his family physician about post-biopsy bleeding; there was no evidence that this man actually experienced an infection.

### Issues covered by interview topic guide

- **Expectations of biopsy** – *understanding of purpose, procedures, comments on information received*
- **Experience of biopsy** – *physical/psychological – what could have been improved in procedures or information*
- **Effects of biopsy** – *physical/psychological/sexual, duration*
- **Experience of infection** – *symptoms, decision to seek help, treatment, impact, what could have been improved*
- **Recommendations for change** - *procedures or information*
- **Views on repeat biopsy**
*see Avery et al. 2008 [17] *for full range of topics covered in interviews with ProBE men*.

### Analysis

Analysis focussed on interview data relating to topics listed in the interview topic guide. Interviews were audio-recorded, transcribed verbatim and transcripts scrutinized to identify recurrent themes. Interviews were analysed in groups (e.g. according to biopsy outcome, experience of infection, number of biopsies) and individually to identify commonalities and contrasts between groups or individuals. The interview topic guide was refined during the interview process as themes emerged, in accordance with constant comparison methods derived from grounded theory [18,19]. Data collection and analysis were carried out iteratively with analysis guiding further sampling for example including men experiencing infection, until no new findings emerged. Analysis was facilitated by use of qualitative computer software, NVivo [20] and led by JW, with a subset of 33 transcripts analysed independently by JLD. Emergent findings were discussed and synthesised to maximise reliability of thematic coding and data interpretation [18,19].

### Development of proposed biopsy patient information leaflet

Findings from the questionnaire study [11] provided current generalizable quantitative data on the physical sequelae that may be expected in the 35 days following biopsy. Findings from the interview study were combined with these quantitative data to propose a revised set of information to be given to men undergoing TRUS-Bx.

## Results

Interviews were conducted with 38 of 45 men (84%, A1-A38, Table 1) from within ProBE and with 47 of 53 men (89%, B39-B85, Table 1) from within ProtecT. Data from interviews with 85 men with a broad range of characteristics were analysed (Table 1). Findings revealed that pre-biopsy information provision played a key role in determining how men experienced biopsy: how well-prepared they were had potentially more influence than the severity of their symptoms in influencing how they experienced TRUS-Bx and its sequelae.

### Levels of preparedness

Most men (N = 64) reported feeling adequately prepared for TRUS-Bx and described the experience as matching what they expected from the PIL or pre-biopsy discussions:

‘*It was exactly as explained to me in the notes, so you have no recourse’* (B52).

‘*Nothing was a shock, what actually happened, because I was told exactly what happened. What was going to happen did happen, there were no nasty shocks involved’* (A5).

Several commented on the effect of previous experiences of ‘*close inspection’* or painful treatments. Six men believed such experiences (endoscopies, treatment for haemorrhoids, a hip operation, a liver biopsy and spinal surgery) made TRUS-Bx less alarming:

*‘The biopsy was child’s play compared to that (previous endoscopy investigation*)’ (B47).

*‘I’ve had spinal operations and that recently, so nothing tends to faze me’* (A3)

Two men, who had no previous experience of hospital treatment, reported this made biopsy more challenging:

*‘For someone who has really never really been in hospital, it becomes a big thing’* (A4)

A substantial minority (21/85, 25%) reported feeling unprepared in a number of ways relating to experience of blood loss, pain, infective symptoms, aspects of biopsy procedure or terminology and repeat biopsy experiences. Men highlighted the discrepancy in their experience between what they expected and what they experienced in terms of quantity, duration, site, colour and the intermittent pattern of bleeding:

**‘*I had no idea just how much blood would come through…and I think it would be an idea to warn people****…to use a condom…that would save a lot of embarrassment and grief*’ (A4).

**‘*I didn’t expect the blood when I stood up****[post biopsy] because it was a lot happening in the back passage so for it to come out of the penis down your legs was a bit sort of ‘hm!’****I was told it could go on for 48 hours, sometimes a bit longer, but it kept on for about 2 weeks’*.** (B73)

***‘The other thing they didn’t tell you on that [PIL]****is that you when you’ve had the biopsy,****you get blood in your semen, not red blood, black.*’**(A7)

Information failed to prepare men for the range of experiences of pain:

‘*I found it incredibly painful and distressing…in an emotional sense*…*biopsy with a nail gun.****She (nurse) said “some people find discomfort”, I think that was a euphemism’*** (A33)

*‘I felt invaded, it took me to my limit…I’ve had this terrible battering* (B62).

Some (4/85) emphasised the experience of distress associated with the procedure, even when pain was well controlled:

*‘I must admit it was a little more severe than I’d anticipated, I nearly passed out…There wasn’t any actual pain just discomfort when you’ve got a tube rammed up your back (side)’* (A1).

These men emphasised that distress or anxiety was not necessarily linked to severity or duration of symptoms *per se* but arose where there was a disparity between expected and actual experience (see comments highlighted in bold above).

Men experiencing infection reported both alarm at the severity of infective symptoms and simultaneous uncertainty about the appropriate response:

*‘Then within an hour I started uncontrollable shakes and shivering, but felt really hot and eventually I said ‘I can’t play this [board game with friends] anymore I’m going to have to go home’. I got home and I went straight to bed. ((Wife’s name)) read the leaflet on from the biopsy and saw about ‘flu symptoms and stuff like that. So on the Saturday morning she rung the emergency doctor and the doctor said just go straight to [emergency service]. And me being me, I was totally out of it and in bed and she said ‘We’ve got to go to [emergency service]’ and I said ‘Oh I’m not going to any hospital [emergency service].”* (A35i)

*‘My son came from work, and I said ‘Please take me to the hospital because I’m not feeling very well and I’m shivering’. And I hadn’t told him about the biopsy, I hadn’t told him anything, and he said, ‘Don’t be so daft, get into bed like’, you know, as you would. ‘You can’t go to hospital for the shivers, because you’re shivering’. So, I then had to tell him, about the biopsy and things. And, so he took me to the [out of hours’ family physician service].’* (A36i)

One also reported an inappropriate response from the emergency doctor when he presented with post-biopsy infection:

‘*They said at the hospital when you were having the biopsy they said ‘should you get any ‘flu-like symptoms, don’t hesitate to go to the hospital and tell them, tell them what you’ve had [done]’. And I go into [hospital name] on the Sunday afternoon, the first of the doctors, you know, looked in complete amazement at me: ‘What are they sending me people like this for? I’ve got enough problems to deal with.’ (A36i)*

Aspects of TRUS-Bx terminology or procedure caused confusion. Some men were not prepared for ten biopsy cores, expecting ‘*a single prostate biopsy’*.

*‘They took 10 biopsies, which was a lot more than I thought they were going to do, which concerned me a little bit. Ten biopsies, what do they need 10 biopsies for, how big is this thing? You know it’s the size of a golf ball, how the hell do they get 10?’ (A5)*

Others reported fear of TRUS-Bx causing cancer spread, confusion over why they should bring a friend or relative to the appointment, and surprise that staff present were all female:

*‘I wondered about how safe the biopsy procedure is. Whether by firing ten holes into the prostate that doesn’t increase the risk of malignant cells escaping from what I think you call the prostatic capsule....Since I had my biopsy that was now about five weeks ago, for a lot of time I’ve had a sort of discomfort in the lower stomach area.’* (A27)

*‘I didn’t even know if they were going to give me an injection and knock me out…I had a horrible feeling that was what was going to happen if they needed somebody else with me to get me home.’* (A12)

‘*I walked into the doctors surgery and it was a female nurse and a female doctor and they were both good looking girls and you know, there was two other women in there and I’ve no idea whoever they were but they was looking quite busy and I was lying on this bed with my trousers and pants down round my ankles and I’m thinking it’s not the right place for this’*. (B67)

Men undergoing repeat diagnostic TRUS-Bx were not prepared for these seeming more invasive and painful:

*‘I hardly felt any discomfort with the first one, it was over in no time at all, just slightly, slightly unpleasant. The second one took a lot longer, had a lot of bleeding, took longer, far more invasive and I felt, I felt poorly after…sicky discomfort in my lower region and it felt far more invasive than the first one’*. (B50)

*‘But the second one [biopsy procedure] made me jump because, I don’t know whether with the first one they kind of took the edge of it and the second time it went right through the middle and it certainly made me go “ooh”’.* (B49)

### Impact of unanticipated TRUS-Bx experiences

Failure to prepare men adequately for TRUS-Bx or its sequelae had a number of consequences. The most common was anxiety (see also Wade et al. [12] who found that men who experienced symptoms as problematic had raised anxiety). This in turn prompted some men to consult health professionals for advice (see also Rosario et al. [11] who reported 119/1147 or 10.4% men contacted health professionals for advice in this way):

*‘The danger is if you say to people that [it] could bleed up to a fortnight, that’s what your little mind tells you. But a week after that, I started bleeding again, so I had to phone my doctor’*. (B62)

*‘But when we got to week four or five and I was still noticing that [blood in urine] then I made the phone call to ask is this as it should be. For some men to find that they were experiencing blood stains….for a period afterwards, would worry some people more than perhaps me*’. (B52)

In most cases (i.e. where there were no infective symptoms) men were simply reassured that symptoms were normal. Men reported frustration at not being adequately prepared, as it would have avoided both unnecessary anxiety and consultations:

*‘It wouldn’t be bad if they were advised that this is something that they should anticipate’.* (B52)

*‘I wish they would have told me that, at the start you know’ (A28)*

These views contrasted with those whose experiences conformed to information provided.

*‘I was well advised as to what would happen on the blood in the urine and blood in the semen and that sort of thing. And it wasn’t then unexpected. I was quite well prepared. And, true to form, they said, you know, it’s sort of three to four weeks or whatever it’ll dissipate. Which it did. And so I was well advised, and I’ve no complaints whatsoever on that score.’ (B65)*

The biopsy experience influenced subsequent PCa treatment decision-making or management for some. Four of the five men experiencing post-biopsy infection reported reluctance to undergo repeat TRUS-Bx:

*‘I wouldn’t hesitate because of the procedure. What I would hesitate would be the possibility of infection and the treatment you get when you get an infection’. (A36i).*

*‘But I did say that I would not have that [biopsy] done again, because of, because I caught the infection. The actual [biopsy] itself um, as I said was quite uncomfortable, but I mean I could manage that. But it was the, the infection afterwards, that, that done it for me really.’ (A37i)*

One man refused radical prostatectomy (RP) treatment because his experience of longer bleeding than described in the information convinced him he would experience more severe side-effects than outlined in prostatectomy information:

*‘But the worrying aspect that I found, was that I had read literature which led me to expect 24 to 48 hours [bleeding]. Several days and it was running into the weekend after I had had the biopsy, I was still passing up quite a lot of blood, so I rang up the emergency doctor cover at the weekend to get some advice and the advice from the doctor was he would give me some more antibiotics…then I thought, ah now, if in fact my post-surgery experiences reflect my post biopsy experiences then the expectation of a relatively quick recovery or recovery at all might be quite prolonged’.*

### How information provision might be improved

Men, who felt prepared, reported that clinical staff had not only provided the PIL but also talked through the procedure and gave opportunities to ask questions and clarify misunderstandings:

‘*The staff in the hospital I had the biopsy, they explained all this to me. So I wasn’t scared when I seen this blood. It’s what I expected.’* (B70).

‘*Everything to me- for me - was explained explicitly before and I was told what to expect, so by the time I went in for the biopsy, I knew exactly what was going to happen…so everything worked like clockwork you know*.’ (B51)

Men suggested that pre-biopsy information they received had been too conservative regarding the range of experience they should have expected. Recommendations for improvements to patient information about TRUS-Bx are summarised in Table 2. Proposed content for a more comprehensive TRUS-Bx PIL, combining current quantitative data from the ProBE questionnaire study [11] and insights from the present study, is presented in Additional file 2.

**Table 2:** **Summary of information that men suggested should be added to patient information leaflets**

**Topic**	**Additional information suggested**
**Pain/Soreness**	
-Intensity	Some men report experiencing intense pain and/or distress during biopsy.
-Duration	Pain and soreness may last up to 4–6 weeks after biopsy.
**Bleeding**	
-Site of bleeding	There may be considerable blood loss immediately following biopsy from the penis, in the semen, urine and from the back passage.
-Quantity of blood loss	The amount of blood in the semen, urine and back passage may appear enough to mask the semen/urine/faeces.
-Duration of bleeding	Bleeding may last (or occur intermittently) for 5 weeks after biopsy.
-Stop/start bleeding	Bleeding may stop after a fortnight, and then restart e.g. a week later.
-Appearance of blood	Blood may appear red or black in colour, may appear as clots or solid lumps or as fluid.
**Biopsy procedures**	
−10-12 cores taken	Normally between 10 and 12 biopsy samples will be taken and each sample requires the needle to be fired into the prostate
-Anaesthetic	Men will be given a local anaesthetic, injected into the prostate. Occasionally, men feel faint after biopsy and this is why men are asked to bring someone to drive them home afterwards.
-Staff present	Biopsy will be carried out by an urologist, radiologist or specialist nurse and a nurse will also be present. Male and female members of staff may be present.
**Fear of cancer spread**	No evidence has been found that biopsy can result in cancer spreading more quickly.
**Fear of hospital acquired infection**	The risk of hospital acquired infection is:
	• Colonisation with MRSA (0.9% - 1 in 110)
	• Clostridium difficile bowel infection (0.01% - 1 in 10,000)
	• MRSA bloodstream infection (0.02% - 1 in 5000)

Other comments from men:

Men who felt well-prepared, reported that health professionals had talked through the procedure with them before biopsy.

Men who had undergone previous invasive medical procedures felt less concern about biopsy than those who had not.

## Discussion

This qualitative study revealed that anxiety associated with TRUS-Bx arose most commonly when experiences or symptoms deviated or were more severe than described in pre-biopsy information. Men responded to anxiety by contacting health professionals for reassurance and most received reassurance rather than active treatment. Men suggested that improvements to pre-biopsy information provision were needed. Given that the prevalence of any haemorrhagic or infective symptoms in the 35 days following TRUS-Bx is known to be 94.0% and in 27.1% symptoms were problematic [11], pre-biopsy information must reflect this if it is effectively to help men manage known symptoms of biopsy, reduce anxiety and avoid unnecessary consultations. Based on these figures and the findings from this interview study, we propose the inclusion of additional pre-biopsy information that more accurately reflects the prevalence and severity of symptoms and that should more effectively prepare men.

This study addressed questions raised by previously published work from the ProBE study: what caused around one quarter of men to experience TRUS-Bx symptoms as problematic [11]; and why those who experienced symptoms as problematic also experienced raised anxiety [12]. This interview study revealed both the influence of information provision in determining how biopsy and its sequelae are experienced and the impact of this on healthcare contact following biopsy. Most of those seeking healthcare contact did so because of unexpected haemorrhagic symptoms and in fact required no active treatment. In contrast, the small number of men experiencing post-biopsy infection reported considerable reluctance to seek healthcare and one man reported an inappropriate response from healthcare professionals. A recent systematic review of biopsy complications highlighted that although minor bleeding and urinary complications are relatively common, these usually require no intervention, whereas serious infective complications though rare, require prompt intervention [4]. Information provision is essential in enabling men to discriminate between minor and serious infective complications. Evidence also suggests that information provision impacts on well-being and health outcomes [21,22]. Men in this study suggested that optimum information provision included face-to-face discussion with a specialist nurse or clinician before TRUS-Bx. Previous qualitative investigations of TRUS-Bx have highlighted emotional costs and anxiety reported by men undergoing biopsy [17,23-26], and a link between information provision and anxiety has been proposed [17,24,26]. Men need time before or after TRUS-Bx to talk through individual fears and misconconceptions [24,9]: concerns and misconceptions raised by men in the present study were highly individual and written PILs cannot anticipate all concerns.

One quarter of this interview study sample reported feeling unprepared in some way, a figure comparable to the 27.1% reporting one or more symptoms as problematic during the 35 days post-biopsy [11]. Symptoms were understated in the sample PIL provided by one ProBE study centre (Additional file 1), when compared to symptoms reported in the main ProBE study findings [11] and those reported here and issues that caused misunderstanding (Table 2) were not all covered (Additional file 1). Key insights into men’s experience of biopsy and its sequelae have therefore been combined with quantitative findings from the ProBE study regarding prevalence of symptoms [11], to propose comprehensive information to better prepare men for TRUS-Bx (Additional file 2). Delayed or inappropriate responses to infective symptoms could be avoided by providing men undergoing prostate biopsy with a card that details the date and location of the procedure, antibiotic cover used, and outlining symptoms that should trigger prompt healthcare consultation. Urologists should also be aware of the potential impact of adverse biopsy experiences on later decisions about re-biopsy or treatment, particularly as more men consider AS, which includes scheduled re-biopsy, as a treatment option for localised PCa.

Recent debate in the UK has criticised variation in existing patient information for TRUS-Bx and called for a systematic approach to patient information whereby information is evidence-based, produced in consultation with patients and whereby the impact of information is measured [22,27,28]. The proposed TRUS-Bx PIL developed in this study is the first step in this process, combining latest evidence from the ProtecT and ProBE studies in accordance with recently published UK guidelines on PCa [10]. It will need evaluating, taking into account local patient and professional views and tested in different languages if used outside the UK.

The main strength of this study was its use of a qualitative methodology enabling exploration and mapping of how and why anxiety arose, and how it prompted unnecessary contact with health professionals. It also benefitted from being embedded in the wider prospective quantitative investigation of the side effects of a systematically applied prostate biopsy protocol and recruited an unusually large sample for an in-depth interview investigation. Limitations include that, as some time has passed since these data were collected, TRUS-Bx techniques and information will have evolved. However, elements of the procedure that took men by surprise (number of biopsy cores, repeat biopsy feeling more invasive) continue to be relevant and are not always covered in current biopsy information. For some participants, interviews took place several months after biopsy and, for some, following radical treatments for PCa. However, data collection in the ongoing ProtecT study [16] has shown that long time intervals between biopsy and interview have not resulted in change to men’s narratives, with both negative and positive experiences of biopsy continuing to be recounted in the same terms, many years after biopsy. Participants in the ProtecT/ProBE study are mostly Caucasian and it should be taken into account that different ethnic groups may report different experiences. Data on interviewees’ education status were not collected, yet education status can influence understanding of and responses to information. The study took place in a research rather than clinical setting and the proposed patient information will need testing for its acceptability and effectiveness in routine care and by participants with a range of education status and from a range of socioeconomic backgrounds. However, given that information provision is likely to receive higher priority in a research than in a non-research setting where additional consent processes are required, it can be assumed that similar issues around information provision would be more likely to arise in a non-research setting. The proposed information is intended for men who have already undergone initial PSA testing. Previous research suggests that information provision may influence men’s decisions whether to present for PSA testing or undergo biopsy [29] and this information will need adapting for men deciding whether to initiate PSA testing. Template or magnetic resonance imaging (MRI) guided biopsy are becoming increasing common and will have a different profile of side effects. However, the key findings of this study (the impact of providing both comprehensive information and time for discussion prior to biopsy on subsequent healthcare contact) are equally applicable to novel procedures. Future research is needed to investigate the finding that repeat biopsies were experienced as more painful and invasive.

## Conclusions

This study illuminated the experiences of men undergoing TRUS-Bx and highlighted that men experienced anxiety associated with biopsy particularly if they were inadequately prepared for the procedure or its after-effects. Men’s experiences have been used to propose a revised TRUS-Bx PIL and reiterate the need for discussion with a specialist nurse or clinician prior to undergoing TRUS-Bx. This approach has potential to reduce anxiety, avoid unnecessary healthcare consultations and facilitate rapid consultation where appropriate.

## Additional files

Additional file 1:
**Content of local trust patient Information leaflet given to men at one ProBE study centre.**
Additional file 2:
**Proposed content for patient information leaflet for men undergoing Transrectal Ultrasound (TRUS) guided prostate biopsy.**

## Abbreviations

AS: Active surveillance
EAU: European Association of Urology
HRQOL: Health related quality of life
MRI: Magnetic resonance imaging
PCa: Prostate Cancer
PIL: Patient information leaflet
ProBE: *Pro*state *B*iopsy *E*ffects study
ProtecT: *Pro*state *te*sting for *c*ancer and *T*reatment trial, International Standard Randomised Controlled Trial Number 20141297
PSA: Prostate specific antigen
RCT: Randomised controlled trial
RP: Radical prostatectomy
TRUS-Bx: Transrectal ultrasound guided biopsy
