# Supplementary material for: Role of information in preparing men for transrectal ultrasound guided prostate biopsy: a qualitative study embedded in the ProtecT trial
Source: BMC Health Serv Res. 2015 Feb 28;15:80. doi: 10.1186/s12913-015-0729-z (PMC4350900; doi:10.1186/s12913-015-0729-z)
Supplement: Additional file 1: — Content of local trust patient Information leaflet given to men at one ProBE study centre. [file 12913_2015_729_MOESM1_ESM.docx]

**Figure S1 Content of local Trust Patient Information Leaflet given to men at one ProBE study centre.**

**Trans Rectal Ultrasound Scan and biopsy of the prostate gland (TRUS)**

**Department of Urology**

**What is the prostate gland?**

The prostate gland is part of the male reproductive system; it produces a fluid to transport semen. It is about the size of a walnut and lies just below the bladder. The tube that drains the bladder passes through the middle of the prostate, and is called the urethra. Immediately behind the prostate is the back passage or rectum. To examine the prostate gland, the doctor will insert a gloved and lubricated finger into the rectum; this allows them to feel the size and shape of the prostate gland. Often it is necessary to have a more ‘in depth’ look at the prostate and requires a special test called a trans-rectal ultrasound scan or TRUS.

**What is a TRUS?**

A tube shaped object, known as a probe, is inserted into the rectum. This is about four inches in length and one inch in diameter with a handle housing the control buttons. It is well lubricated to aid its insertion. Once in place, sound waves echo against the prostate to produce an image on the screen. This allows the doctor to look at the size and shape of the gland. A local anaesthetic is injected through the probe at this point to reduce any discomfort. Cancer in the prostate cannot always be seen, as it is microscopic, therefore we will take some samples of tissue with a needle that passes through the middle of the probe, this is known as a biopsy. The biopsy needle is on a spring-loaded mechanism and makes a loud crack as it is fired; please do not be alarmed. The sensation is described as more of a discomfort than pain. As these are very small pieces of tissue it is necessary to take enough for a doctor in the laboratory to work with, we take an average of ten biopsies.

**What preparation will I need for the test?**

No special preparation is needed for this test; however, it might be more comfortable for you if you are able to empty your bowels before you come to the hospital. Don’t worry if this is not possible. You may eat and drink as normal. We do advise that you might like to arrange for someone to drive you home following the test.

**What is involved in the test?**

After reporting to the receptionist, you will be collected by a nurse and shown to an area where you will be asked to change. This is just a precaution to protect your clothing from soiling. The nurse will explain the procedure to you and ask you a few questions about your health. You will then be shown into the procedure room. The doctor will again explain the procedure to you and he may ask you to sign a consent form. Before the scan the doctor will examine you, take a further sample of blood and give you an injection of antibiotics. The antibiotics are all done through a small butterfly shaped needle in the arm and are given to try to prevent any risk of infection caused by taking the biopsies through the rectum. You will then be asked to turn onto your left side and to tuck your knees up to your tummy, this position allows the probe or doctors finger to enter the rectum in the same direction as the curve of the bowel. The probe will then be inserted and the scan commenced, we will inject the local anaesthetic and may measure the size of the prostate. We will warn you when we are about to take the biopsies. This procedure takes about 10-15 minutes and a nurse will be with you at all times.

**What happens after the test?**

After the scan we will allow you a short rest before taking you back to the changing area. You will be given a small pad to line your underpants with; this will prevent soiling of your clothes as you may pass some blood or some of the lubricant jelly from your rectum. We will also give you a jug to pass urine into; this is to ensure that the test has not stopped you being able to urinate and to assess the level of bleeding you might experience. It is quite common for you to see some blood in the urine, semen and stools following this test, this usually settles but may take up to six weeks. You may help it to clear by drinking plenty.

**Are there any side effects after the test?**

Yes, first there is the side effect of passing blood in your urine, semen and stools, as we have said already this will lessen with time. Occasionally it becomes difficult to pass urine; this is usually because the blood forms a clot in the urethra preventing you from urinating. If this happens you should contact either your GP or this department, where advice and help can be given. Infection is also a risk of this procedure, and while every effort has been made to try and prevent this by the giving of antibiotics you should be aware of this risk. If you have a temperature, pain, persistent burning when passing urine, heavy bleeding or flu-like symptoms then please contact your GP or this department on the contact numbers provided. Your GP will be notified of your attendance for this procedure.

**What do I need to do before I leave the Urology department?**

- To have passed a clear sample of urine.
- To have the opportunity to have drink and recover.
- To be given a letter for you GP.
- To be given a contact card.
- To be given a course of antibiotic tablets or a prescription to collect from the pharmacy.
- To be given an appointment or advice about receiving my results.

**Will I get a clinic appointment?**

Following the test, the sample of tissue will be sent to the laboratories to be examined under a microscope and the results will be reported to your Consultant. This process usually takes approximately three weeks. An appointment to attend a clinic or a letter advising you of your results will be sent to you through the post. If you have any difficulties attending or you are worried that we have forgotten your appointment or letter, please do not hesitate to contact us on the numbers supplied.
