# Supplementary material for: Role of information in preparing men for transrectal ultrasound guided prostate biopsy: a qualitative study embedded in the ProtecT trial
Source: BMC Health Serv Res. 2015 Feb 28;15:80. doi: 10.1186/s12913-015-0729-z (PMC4350900; doi:10.1186/s12913-015-0729-z)
Supplement: Additional file 2: — Proposed content for patient information leaflet for men undergoing Transrectal Ultrasound (TRUS) guided prostate biopsy. [file 12913_2015_729_MOESM2_ESM.docx]

**Appendix 2** Proposed content for patient information leaflet for men undergoing Transrectal-Ultrasound (TRUS) guided prostate biopsy

**Prostate biopsy *or Transrectal Ultrasound Guided Biopsy of the Prostate gland (TRUS-guided biopsy)***

This information leaflet is for men who are waiting for a prostate biopsy, which is also known as TRUS-guided biopsy.

**Where does this information come from?**

We asked over 1,000 men to tell us about their experience of prostate biopsy in a questionnaire. We also interviewed 85 men about their biopsy experience. The information in this leaflet is based on what those men told us.

**What is the prostate? What does it do?**

Only men have a prostate gland. The prostate is a small gland usually around the size and shape of a plum. It lies below the bladder and surrounds the tube (the urethra) that men pass urine and semen through. It can be examined by a doctor putting a finger in the back passage (rectal examination).

**What are the most common prostate problems?**

The three most common prostate problems are an [**enlarged prostate**](http://prostatecanceruk.org/information/enlarged-prostate), [**prostatitis**](http://prostatecanceruk.org/information/prostatitis) and [**prostate cancer**](http://prostatecanceruk.org/information/prostate-cancer).

- An **enlarged prostate** (also known as Benign Prostatic Enlargement – BPE or Benign Prostatic Hyperplasia - BPH): this is the most common prostate problem and means non-cancerous enlargement of the prostate gland.
- **Prostatitis**: this can be caused by either an infection or an inflammation of the prostate. It is not a form of cancer

• **Prostate cancer**: this is when cells in the prostate start to grow in an uncontrolled way. In the UK, about 1 in 8 men (13%) will get prostate cancer at some point in their lives.

**Why do I need a prostate biopsy?**

Your doctor may recommend you have a prostate biopsy, if you have one of the following:

- A raised Prostate Specific Antigen (PSA) test: this is a blood test that measures the total amount of prostate specific antigen in your blood,
- An abnormal feeling prostate gland on rectal examination,

The only way to find out whether there is cancer in the prostate is to take small samples of tissue (biopsies) from the prostate gland and for a specialist doctor (pathologist) to look at these under a microscope.

**What should I do if I take warfarin?**

Please tell the nurse or doctor if you take warfarin. If you take warfarin, you will normally be asked to stop taking it for 3 days before the biopsy. You will need a blood test (INR) at least one hour before your biopsy.

You will need to ensure that any bleeding has stopped for at least 24 hours before you restart taking your warfarin after the biopsy. If you are not sure, check with your doctor or use one of the contact numbers below.

**What should I do if I take other anticoagulants?**

If you take:

- Asprin
- Clopidogrel (plavix)
- Dipyridamole

Tell the nurse or doctor and they will advise you.

**What does a TRUS-guided prostate biopsy involve?**

Prostate biopsy involves taking small samples of tissue from your prostate using a needle that passes through the wall of your back passage (rectum) into your prostate. Prostate biopsies are usually done at an out-appointment in the Urology department.

You will be asked to change out of your clothes into a hospital gown and give a swab from your nose and throat to find out whether you are carrying an infection (MRSA). The doctor will give you an antibiotic injection into your arm to prevent infection from the biopsy.

You will be asked to lie on your left side. The doctor carrying out the biopsy will show you the ultrasound probe and demonstrate the clicking noise it makes. The ultrasound probe is then placed inside your back passage (rectum). The probe gives the doctors an image of your prostate on screen to guide them when taking the tissue samples.

A needle is inserted into your back passage (rectum) alongside the probe and local anaesthetic is injected into each side of the prostate. Once the anaesthetic is working, biopsy samples will be taken.

The biopsy device inserts a needle into your prostate. This needle removes small tissue samples to be sent for analysis under microscope. Between 10 and 12 samples of tissue are normally taken. This takes between 10 and 15 minutes. Most men find the biopsy causes only mild discomfort. If you feel pain that concerns you at any time, you can tell the doctor or nurse or raise your hand.

Prostate biopsy will be done by a urologist, a radiologist or a specialist nurse. A nurse will always be with you during the biopsy. Both male and female staff may be present. It is not possible to say whether cancer is present or not until samples have been examined in the laboratory.

**What happens afterwards?**

We will ask you to stay in the out-patient department until you pass urine. It is quite common to see blood in your urine, semen and stools after biopsy. You will be given some antibiotic tablets to take at home, to prevent infection in the prostate, the urine or the blood. It is important that you take all the antibiotics. Please let the nurse know if you have any allergies to antibiotics.

Your family doctor will be told you have had a prostate biopsy.

**Should I bring a friend or relative?**

Yes, if possible, bring someone with you to drive you or accompany you home. You should not drive yourself because occasionally men report feeling dizzy immediately afterwards. This usually passes within an hour or two.

**Can I eat and drink as normal before and after the biopsy?**

Yes, it helps if you drink plenty of fluid in the first two days after biopsy. You should ask your doctor whether you need to avoid alcohol because of the antibiotics.

**What are the chances of my biopsy result showing I have cancer?**

About one in three men, who have a prostate biopsy because of a raised PSA blood test result (PSA ≥3ng/mL), will be found to have prostate cancer. There may be cancer in your prostate, but this is not shown on your first biopsy. If your biopsy shows no cancer, but you still have a raised PSA in blood tests, you may be offered another biopsy.

**What are the possible side effects?**

Most men having the biopsy feel some discomfort. A small number (1 or 2 out of 10) feel severe pain or distress during biopsy. It is important that you tell the doctor doing the biopsy if this happens, as they may be able to control the pain better.

Almost all men (9 out of 10) will notice blood in their urine, motions or semen after biopsy. This may continue for up to 5 weeks after biopsy. Bleeding may stop, and then start again. This does not mean there is a problem. If the bleeding becomes very heavy or continues beyond 6 weeks, you should contact your doctor.

Very rarely (1 or 2 out of 100), men find it more difficult to pass urine after a biopsy and may need a catheter inserted to drain the bladder.

There is a risk of getting an infection after the biopsy (4 out of 100 men†). The symptoms include pain, shivers, fever and feeling hot and cold. This can still happen even when you take all of the antibiotics you have been given to help prevent this. You are more likely to develop an infection, if you do not take the antibiotics. Very rarely (1 or 2 out of 100) men need to be admitted to hospital to treat the infection.

**(†** Loeb S, van den Heuvel S, Zhu X et al. Infectious complications and hospital admissions after prostate biopsy in a European randomized trial. Eur Urol 2012;61:1110–4)

**Is there a risk that by having a biopsy this will enable the cancer to spread?**

There is no evidence that having a biopsy will allow the cancer to spread.

**What can I do in the days after biopsy?**

You can take simple painkillers to help with any discomfort in the prostate area. You should make sure you take your entire 3-day course of antibiotic tablets. You should drink as much fluid as possible for the first 48 hours after the biopsies. You can eat normally.

It is important that you take it easy and avoid physically demanding tasks for at least two days after biopsy.

You may find blood in the sperm (ejaculate) and because of this some men prefer to use a condom for sex in the first month after biopsy.

**What are the chances of catching a hospital acquired infection as a result of the biopsy?**

You should ask for the local figures for your hospital if you are concerned about this.

**When will I get the results of the biopsies?**

You will be given an appointment to come back to the hospital for your results about 2 weeks after biopsy. This appointment will be given to you at the time of your biopsy.

**Who should I contact if I have problems before the biopsy?**

If you require any further information about this procedure, please contact:

**Urology outpatients**

Floor location and name of building, Name of Hospital

**Nursing Enquiries**

Tel number, Monday to Friday - 8.30am to 5.00pm

**Appointment Enquiries**

Tel number, Monday to Friday - 10.00am to 3.00pm

**After biopsy, you should contact your GP immediately if you have**

- a fever or shivers
- severe bleeding
- severe pain on passing urine or are unable to pass urine

If these problems start outside of GP hours, phone the emergency number for your GP. Tell the doctor that you have had a prostate biopsy and have developed problems.

Details you will need:

• Your appointment letter

• Your hospital number

• Your date of birth

Websites of interest for further information:

www.nhs.uk (NHS choices)

[www.prostatecanceruk.org](http://www.prostatecanceruk.org)

www.**cancers**creening.nhs.uk/**prostate**

**Men’s experiences of prostate (TRUS) biopsy**

We asked over 1000 men to tell us in a questionnaire what their experience of prostate (TRUS) biopsy had been. This box summarises their responses.

**Immediate effects**

• 85% of men described no pain or mild pain only associated with the biopsy procedure.

• 3% of men felt light headed or dizzy after the biopsy

• 7% passed blood in their urine immediately after biopsy

• 3% passed ‘clots’ in their urine immediately after biopsy

**Delayed effects (within 35 days of biopsy)**

• 44% (1 in 2) had pain • 7% (1 in 15) found this a moderate or serious problem

• 20% (1 in 5) had a fever • 5% (1 in 20) found this a moderate or serious problem

• 66% (2 in 3) had blood in the urine • 6% (1 in 16) found this a moderate or serious problem

• 37% (1 in 3) had blood in the motions • 2% (1 in 50) found this a moderate or serious problem

• 90% (9 in 10) had blood in the semen • 25% (1 in 4) found this a moderate or serious problem

Men who experienced symptoms as being a ‘moderate’ or ‘serious’ problem also reported feeling more anxious than men who experienced problems as ‘not a problem’ or ‘a minor problem’.

**Duration of symptoms**

• 15% (1 in 7) of men had any pain lasting for 2 weeks or more after the biopsy

• 3% (1 in 30) had a fever lasting for 2 weeks or more after the biopsy

• 20% (1 in 5) had blood in their urine lasting 2 weeks or more

• 5% (1 in 20) had blood in their motions for 2 weeks or more after their biopsy

• 60% (2 in 3) had blood in their semen for 2 weeks or more after their biopsy
